# Baseline cardiac function checkup in patients with gastric or breast cancer receiving trastuzumab or anthracyclines

**DOI:** 10.1002/cam4.4929

**Published:** 2022-06-11

**Authors:** Taisuke Ishii, Tomone Watanabe, Takahiro Higashi

**Affiliations:** ^1^ Division of Health Services Research National Cancer Center Tokyo Japan

**Keywords:** breast cancer, gastric cancer, ultrasound echocardiogram

## Abstract

**Background:**

Although trastuzumab and anthracyclines are frequently used to treat breast cancer (BC) and gastric cancer (GC), cardiotoxicity is a serious concern. The cardiac function assessment is recommended at baseline before initiating treatment. However, the prevalence rates of baseline cardiac checkups are unknown.

**Methods:**

The national database of hospital‐based cancer registries linked to the health services‐utilization data was used to study patients with newly diagnosed stage IV BC and GC (*n* = 6271) who received trastuzumab (*n* = 4324, 69.0%) or anthracyclines between January 2012 and December 2015. The baseline ultrasound echocardiogram (UCG) performance rate and factors related to adequate UCG performance for all patients and those receiving trastuzumab were analyzed.

**Results:**

The adequate baseline UCG checkup rate was higher in patients treated with trastuzumab than in those treated with anthracyclines (71.8% vs 44.1%, respectively). Additionally, patients with GC were less likely to receive an adequate baseline UCG performance than those with BC (70.4% vs 75.0%, respectively). After adjusting for potential confounders, patients with anthracycline‐treated BC and GC were less likely to receive adequate baseline UCG performance than those with trastuzumab‐treated BC (odds ratio [OR]: 0.24, 95% confidence interval [CI]: 0.20–0.28, and OR: 0.07, 95% CI: 0.03–0.16, respectively). Furthermore, patients with trastuzumab‐treated GC were less likely to receive adequate baseline UCG performance than those with BC (OR: 0.65, 95% CI: 0.50–0.84).

**Conclusions:**

The baseline UCG was less likely to be performed in patients receiving anthracyclines than in those receiving trastuzumab, as well as in patients with GC than in those with BC.

## Graphical abstract

The proper baseline cardiac function checkup was less likely to be performed in patients receiving anthracyclines than in those receiving trastuzumab, as well as in patients with gastric cancer than in those with breast cancer. Interventions for these vulnerable patients are required to improve the quality of care.

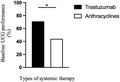

## 1 INTRODUCTION

Trastuzumab is a well‐known monoclonal antibody targeting human epidermal growth factor receptor 2 (HER2)^1^ and was one of the first targeted therapies to show efficacy across various cancer types.^2^ Based on pivotal trials, it was licensed in Japan for metastatic HER2‐positive breast cancer (BC) in 2001^3^ and for metastatic HER2‐positive gastric cancer (GC) in 2011.^4^

Although trastuzumab is well tolerated, cardiotoxicity is one of its most serious adverse effects. The trastuzumab‐induced cardiotoxicity was reported in 4.7% of patients with metastatic BC^5^ and 5.1% of patients with GC.^6^ Although older age and previous anthracycline exposure are well‐recognized risk factors for trastuzumab‐induced cardiotoxicity,^5^, ^7^ other risk factors have been suggested, including hypertension, smoking, preexisting diabetes mellitus, and elevated cardiac biomarkers.^8^ Additionally, baseline left ventricular ejection fraction (LVEF) is linked to trastuzumab‐induced cardiotoxicity.^9^, ^10^ Therefore, the European Society of Cardiology (ESC) recommended assessing cardiovascular risk factors, particularly LVEF, at baseline before starting trastuzumab.^11^ Furthermore, anthracyclines, well‐known traditional chemotherapeutics that also cause cardiotoxicity, are used for metastatic BC as well as early BC^12^ and are classically used for metastatic GC.^13^ Because preexisting cardiac disease is considered a risk factor for anthracycline‐related cardiotoxicity, the ESC also recommended assessing cardiac function at baseline before starting anthracycline.^10^

To the best of our knowledge, no large‐scale data have yet been used to evaluate the current status of proper baseline cardiac function checkups and related factors in patients with metastatic BC or GC who received trastuzumab. Furthermore, discrepancies in the performance of baseline cardiac function checkups between trastuzumab and anthracyclines have not been investigated. This study investigated whether the performance of a baseline cardiac function checkup before starting cardiotoxic systemic therapy was related to the type of systemic therapy (trastuzumab vs anthracyclines) and cancer type (BC vs GC) among patients with advanced‐stage disease, because trastuzumab and anthracyclines, unlike BC, could only be used for patients with advanced GC stage.

## 2 METHODS

### 2.1 Study population

This study assessed data from adult patients with newly diagnosed stage IV BC and GC who were treated at hospitals participating in a large quality‐of‐care monitoring project between January 2012 and December 2015.^14^ These patients were classified into four categories: (1) patients with BC who received trastuzumab but did not receive anthracyclines before trastuzumab initiation; (2) patients with GC who received trastuzumab but did not receive anthracyclines before trastuzumab initiation; (3) patients with BC who received anthracyclines but did not receive trastuzumab before anthracycline initiation; and (4) patients with GC who received anthracyclines but did not receive trastuzumab before anthracycline initiation. The treatment initiation timeframe was specified from October 2011 to December 2017. Anthracyclines are defined as either doxorubicin or epirubicin.

### 2.2 Data sources

This study was conducted as a part of the quality‐of‐care project approved by the Institutional Review Board of the Japanese National Cancer Center. The acquisition of data for the quality‐of‐care monitoring project has been described in detail elsewhere.^15^ Briefly, the health services‐utilization data corresponding to health insurance claims were collected from the Diagnosis Procedure Combination (DPC) survey and were linked to the National Database of Hospital‐Based Cancer Registries (HBCR). Although DPC survey data are not used for reimbursement, they do include information about all billable health care services provided, including prescription medications, as well as the specialty of the physicians who provide the service in a manner similar to fee‐for‐service insurance claims. The HBCR is a compulsory reporting system for patients with newly diagnosed cancer in the Japanese Ministry of Health, Labor, and Welfare‐designated cancer care hospitals. Additionally, nondesignated hospitals, which play an equivalent role to designated hospitals in their respective communities, have the option to participate in the HBCR. The HBCR contains clinical information data such as age at diagnosis, gender, cancer type, clinical, and pathological stages of cancer, tumor–node–metastasis classifications, histopathological type according to the International Classification of Diseases for Oncology (3rd edition), and hospital type (designated or nondesignated). The HBCR also includes a code that identifies each participating hospital. From October of the year before diagnosis to the end of the year after diagnosis, DPC survey data for patients registered in the HBCR database were collected and linked to the HBCR. The linked DPC survey data were used to identify the performance of ultrasound echocardiograms (UCGs) and the administration of trastuzumab and anthracyclines.

### 2.3 Definitions

According to clinical practice guidelines, an adequate baseline cardiac checkup for patients with stage IV cancer receiving trastuzumab or anthracyclines was identified based on the UCG performance before systemic therapy initiation.^8^, ^11^ Additionally, based on a previous study, the UCG performance before systemic therapy administration was defined as receiving UCG within 100 days before systemic therapy.^16^ Furthermore, the specialty of physicians who initiated trastuzumab or anthracycline therapy for each patient was identified according to the department where each patient received cancer treatment (Internal Medicine or Surgery). In Japan, BC and GC are generally treated by surgical oncologists, gastroenterologists, or medical oncologists. Since surgical oncologists are defined as surgeons, and gastroenterologists and medical oncologists are recognized as internists in Japan, we used this classification in this study.

### 2.4 Statistical analysis

The demographic characteristics of patients who received anthracyclines were compared with those of patients receiving trastuzumab. To assess the factors related to adequate UCG performance, the demographic variables of patients who had or did not receive adequate UCG checkups were compared. Additionally, the demographic variations between patients with BC and those with GC who received trastuzumab were assessed. The Student *t*‐test or Mann–Whitney *U* test was used for continuous variables, whereas the chi‐squared test was used for categorical variables. We used a multilevel logistic regression model to assess factors related to adequate UCG performance in all patients and in those receiving trastuzumab after taking into account differences in the practice patterns of each hospital. In the multivariable analysis, UCG performance was adjusted for age at diagnosis, gender, and treating physicians' specialty (internist vs surgeon), as well as the treatment combination and cancer type, as appropriate (trastuzumab‐treated BC vs trastuzumab‐treated GC vs anthracycline‐treated BC vs anthracycline‐treated GC for all patients and BC vs GC for patients who received trastuzumab). In these multilevel models, the hospital code was used as a second‐level variable. All statistical tests were two‐sided, and all analyses were conducted using the Stata v13.2 software (StataCorp LP). A *p* value of less than 0.05 indicates statistical significance. Graphs were created using the GraphPad Prism 9 software (GraphPad Software Inc.).

## 3 RESULTS

### 3.1 Patient characteristics

In this study, a total of 6271 patients with stage IV BC or GC were included. They were treated at 510 hospitals, 364 (71.4%) of which were designated and 146 (28.6%) of which were not. Trastuzumab was given to 4324 patients (69.0%), and anthracyclines were given to 1947 (31.0%). Patients receiving trastuzumab were older than those receiving anthracyclines (64 vs 56 years, *p* < 0.001, respectively) and more likely to be treated by internists than those receiving anthracyclines (51.4% vs 8.2%, *p* < 0.001, respectively; Table 1). Trastuzumab‐treated patients (*n* = 4324) comprised 1334 patients with BC (30.9%) and 2990 patients with GC (69.1%) treated at 485 hospitals (351 designated and 134 nondesignated hospitals). Patients with GC were older than those with BC (67 vs 59 years, *p* < 0.001, respectively) and more likely to be treated by internists than those with BC (68.8% vs 12.4%, *p* < 0.001, respectively; Table 2).

**TABLE 1:** Characteristics of all patients stratified by anticancer therapy regimen

Characteristics	Total (*N* = 6271)	Trastuzumab (*n* = 4324)	Anthracyclines (*n* = 1947)	*p* value
Age, mean (SD, min–max), years	62 (12, 21–92)	64 (11, 21–92)	56 (11, 22–90)	<0.001
≥ 65, *n* (%)	2795 (44.6)	2337 (54.1)	458 (23.5)	<0.001
Gender (female), *n* (%)	3910 (62.4)	2003 (46.3)	1907 (98.0)	<0.001
Cancer type, *n* (%)				
Breast cancer	3246 (51.8)	1334 (30.9)	1912 (98.2)	<0.001
Gastric cancer	3025 (48.2)	2990 (69.1)	35 (1.8)	
Treatment duration, median (IQR), days	133 (63–318)	208 (77–398)	70 (63–119)	<0.001
Physician specialty, *n* (%)				
Internal medicine	2380 (38.0)	2221 (51.4)	159 (8.2)	<0.001
Surgery	3891 (62.0)	2103 (48.6)	1788 (91.8)	
Hospital type, *n* (%)				
Nondesignated hospital	715 (11.4)	471 (10.9)	244 (12.5)	0.059
Designated hospital	5556 (88.6)	3853 (89.1)	1703 (87.5)	

Abbreviations: IQR, interquartile range; min, minimum; max, maximum; SD, standard deviation.

**TABLE 2:** Characteristics of trastuzumab‐receiving patients stratified by cancer type

Characteristics	Total (*N* = 4324)	Breast cancer (*n* = 1334)	Gastric cancer (*n* = 2990)	*p* value
Age, mean (SD, min–max), years	64 (11, 21–92)	59 (12, 26–91)	67 (10, 21–92)	<0.001
≥65, *n* (%)	2337 (54.1)	420 (31.5)	1917 (64.1)	<0.001
Gender (female), *n* (%)	2003 (46.3)	1326 (99.4)	677 (22.6)	<0.001
Trastuzumab duration, median (IQR), days	208 (77–398)	395 (210–525)	154 (56–302)	<0.001
Physician specialty, *n* (%)				
Internal medicine	2221 (51.4)	165 (12.4)	2056 (68.8)	<0.001
Surgery	2103 (48.6)	1169 (87.6)	934 (31.2)	
Hospital type, *n* (%)				
Nondesignated hospital	471 (10.9)	143 (10.7)	328 (11.0)	0.81
Designated hospital	3853 (89.1)	1191 (89.3)	2662 (89.0)	

Abbreviations: IQR, interquartile range; min, minimum; max, maximum; SD, standard deviation.

### 3.2 Baseline UCG checkup rate and the related factors

**FIGURE 1:**
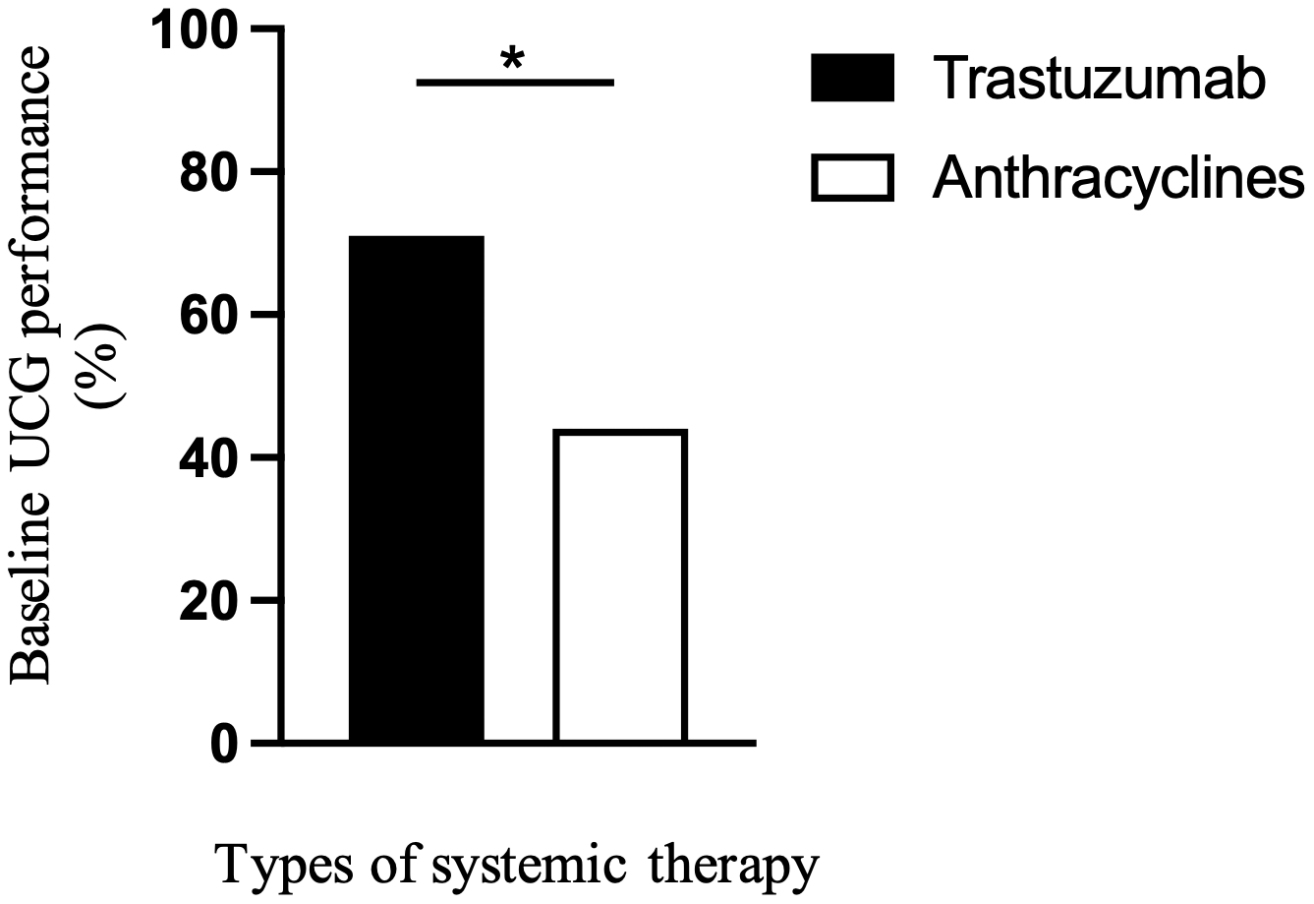
Differences in the baseline UCG performance between patients receiving trastuzumab and those receiving anthracyclines. *Among all included patients (*n* = 6271), patients receiving anthracyclines had an adequate baseline UCG less frequently than those receiving trastuzumab (71.8% vs 44.1%, *p* < 0.001, respectively). BC, breast cancer; GC, gastric cancer; UCG, ultrasound echocardiogram.

Overall, 63.2% of patients treated with trastuzumab or anthracyclines had adequate UCG performance. The unadjusted comparison revealed that patients treated with trastuzumab had an adequate UCG performance rate higher than that of patients treated with anthracyclines (71.8% vs 44.1%, *p* < 0.001, respectively; Figure 1). Stratification based on adequate UCG performance revealed that the adequate UCG group has significantly more patients with GC and more patients treated with trastuzumab, as well as more patients treated by internists (Table S1).

After adjusting for age at diagnosis (<65 vs ≥65 years), gender, treating physicians' specialty (internist vs surgeon), and treatment and cancer type combination (trastuzumab‐treated BC vs trastuzumab‐treated GC vs anthracycline‐treated BC vs anthracycline‐treated GC), we found that patients aged ≥65 years were more likely to receive an adequate UCG performance (odds ratio [OR]: 1.28, 95% confidence interval [CI]: 1.13–1.46, *p* < 0.001), whereas patients who were treated by a surgeon (OR: 0.75, 95% CI: 0.64–0.90, *p* = 0.001), patients with trastuzumab‐treated GC (OR: 0.62, 95% CI: 0.48–0.80, *p* < 0.001), patients with anthracycline‐treated BC (OR: 0.24, 95% CI: 0.20–0.28, *p* < 0.001), and patients with anthracycline‐treated GC (OR: 0.07, 95% CI: 0.03–0.16, *p* < 0.001) were less likely to receive adequate UCG performance (Table 3).

**TABLE 3:** Factors related to UCG performance in all patients (*n* = 6271)

Characteristics	Unadjusted odds ratio (95% CI)	*p* value	Adjusted odds ratio (95% CI)^a^	*p* value
Age (years)				
<65	Reference		Reference	
≥65	1.62 (1.44–1.82)	<0.001	1.28 (1.13–1.46)	<0.001
Gender (female)				
Male	Reference		Reference	
Female	0.63 (0.56–0.72)	<0.001	1.04 (0.85–1.27)	0.70
Treatment and cancer type				
Trastuzumab and BC	Reference		Reference	
Trastuzumab and GC	0.76 (0.65–0.90)	0.001	0.62 (0.48–0.80)	<0.001
Anthracyclines and BC	0.23 (0.19–0.27)	<0.001	0.24 (0.20–0.28)	<0.001
Anthracyclines and GC	0.08 (0.03–0.19)	<0.001	0.07 (0.03–0.16)	<0.001
Physician specialty				
Internal medicine	Reference		Reference	
Surgery	0.54 (0.48–0.62)	<0.001	0.75 (0.64–0.90)	0.001

Abbreviations: BC, breast cancer; CI, confidence interval, GC, gastric cancer; UCG, ultrasound echocardiogram.

^a^ Adjusted for age, gender, treatment and cancer type classifications, and specialty of the treating physicians.

**FIGURE 2:**
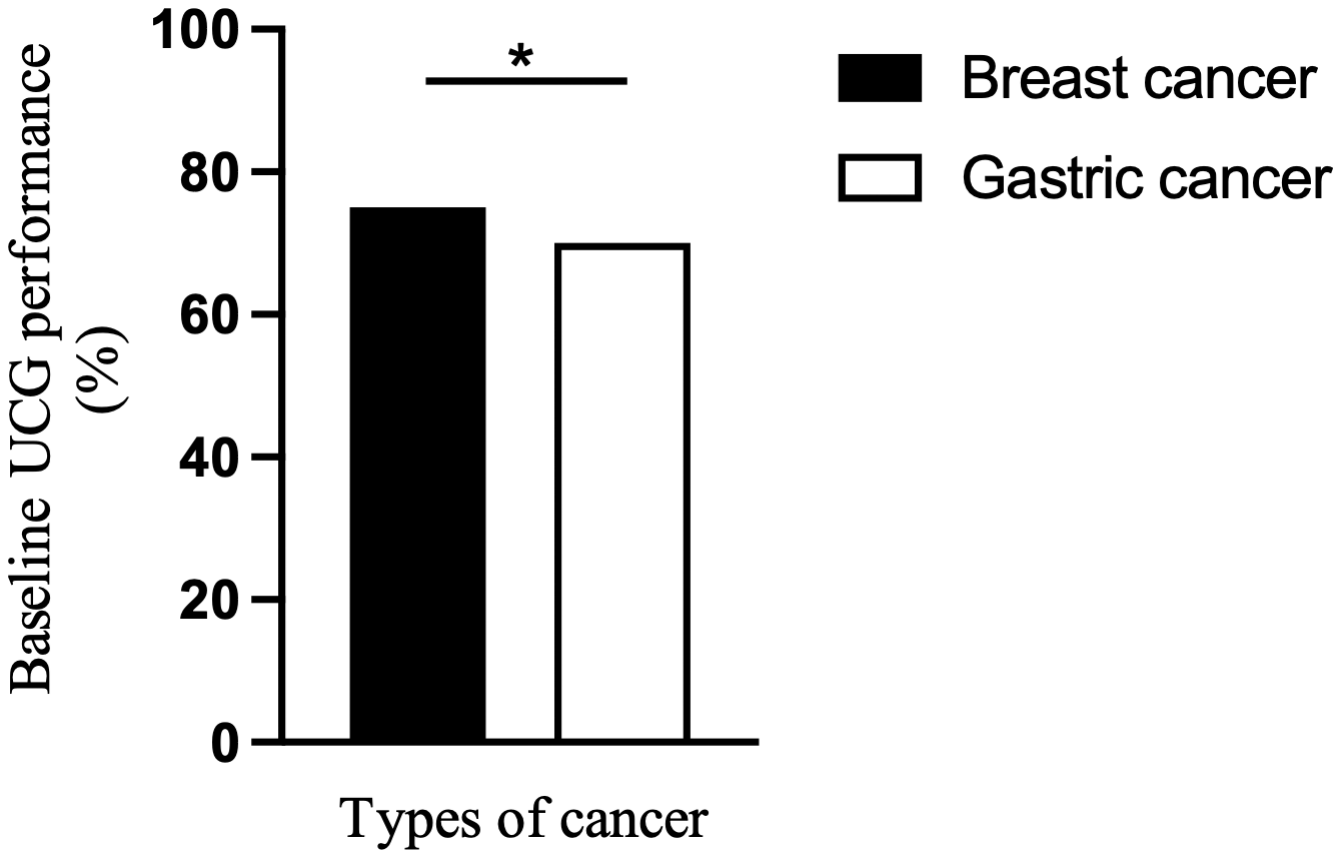
Differences in the baseline UCG performance between trastuzumab‐receiving patients with BC and GC. *Among patients receiving trastuzumab (*n* = 4324), patients with GC were less likely to receive adequate baseline UCG than those with BC (70.4% vs 75.0%, *p* = 0.002, respectively). BC, breast cancer; GC, gastric cancer; UCG, ultrasound echocardiogram.

When we focused our analyses on patients with BC or GC who were receiving trastuzumab, we found that patients with GC were less likely to receive adequate UCG performance than patients with BC (70.4% vs 75.0%, *p* = 0.002, respectively; Figure 2). When patients were stratified by adequate UCG performance, the adequate UCG group had more patients with BC than the inadequate UCG group (32.2% vs 27.4%, *p* = 0.002, respectively; Table S2).

After adjusting for age at diagnosis, gender, treating physician's specialty, and cancer type (BC vs GC), older age was found to be associated with a higher frequency of adequate UCG performance (OR: 1.21, 95% CI: 1.04–1.41, *p* = 0.015; Table 4). By contrast, patients treated by surgeons and those with GC were less likely to receive adequate UCG performance (OR: 0.77, 95% CI: 0.64–0.92, *p* = 0.005 and OR: 0.65, 95% CI: 0.50–0.84, *p* = 0.001, respectively).

**TABLE 4:** Factors related to UCG performance based on a multilevel logistic regression model in patients receiving trastuzumab (*n* = 4324)

Characteristics	Unadjusted odds ratio (95% CI)	*p* value	Adjusted odds ratio (95% CI)^a^	*p* value
Age (years)				
<65	Reference	0.18	Reference	0.015
≥65	1.11 (0.96–1.28)		1.21 (1.04–1.41)	
Gender (female)				
Male	Reference	0.005	Reference	0.49
Female	1.24 (1.07–1.43)		1.08 (0.88–1.32)	
Cancer type				
Breast cancer	Reference	0.001	Reference	0.001
Gastric cancer	0.76 (0.64–0.89)		0.65 (0.50–0.84)	
Physician specialty				
Internal medicine	Reference	0.58	Reference	0.005
Surgery	0.96 (0.82–1.12)		0.77 (0.64–0.92)	

Abbreviation: CI, confidence interval.

^a^ Adjusted for age, gender, cancer type, and specialty of the treating physicians.

## 4 DISCUSSION

Using a large, nationwide database, we revealed that adequate UCG performance was suboptimal in patients with stage IV BC and GC who received trastuzumab or anthracyclines (63.2%). Surprisingly, adequate UCG was less likely to be performed in patients treated with anthracyclines than in patients treated with trastuzumab (44.1% vs 71.8%, respectively). Furthermore, among patients treated with trastuzumab, the adequate UCG performance rate was lower in patients with GC than in those with BC (70.4% vs 75.0%, respectively). It was found that treating GC with anthracyclines is an independent factor related to inadequate UCG performance.

Since it has been known for a long time that anthracyclines can cause cardiotoxicity, we expected that the adequate UCG performance rate before anthracycline initiation would be higher than that before trastuzumab initiation.^17^ However, we found that adequate UCG was less likely to be performed in patients treated with anthracyclines than in those treated with trastuzumab. To the best of our knowledge, the rate of adequate cardiac function checkups has never been reported. Therefore, we were unable to compare our findings with previous studies. It should be noted that UCG performed before or during trastuzumab treatment could have served as a baseline cardiac function checkup for anthracycline therapy in patients who had previously received trastuzumab therapy. However, to rule out potential drug–drug interactions, we restricted our study to patients who received trastuzumab without a history of taking anthracyclines or anthracyclines without a history of receiving trastuzumab. Another possibility is that UCGs were performed after the anthracyclines were started, since cardiotoxicity usually takes time to manifest.^18^ However, we looked at UCG performance before starting systemic therapy because preexisting heart disease or asymptomatic left ventricular dysfunction was a known risk factor for cardiotoxicity.^19^ Moreover, the ESC recommended performing a UCG assessment of left ventricular function at baseline before starting anthracycline to identify potential risk factors.^11^ Therefore, based on our findings, more attention should be paid to UCG performance before starting anthracycline therapy.

We found that patients with GC were less likely to receive adequate UCG performance than those with BC before trastuzumab initiation. However, patients with GC were older than those with BC, and older age is a risk factor for trastuzumab‐induced cardiotoxicity.^10^ Even after adjusting for age and other potential confounders, GC remained an independent predictor of inadequate UCG performance. The incidence rate of trastuzumab‐induced cardiotoxicity in GC was previously reported as 5.1%, which was the same as in BC.^6^ Furthermore, the ESC recommended assessing cardiac function at baseline before starting trastuzumab therapy for all cancer types.^11^ This difference in practice between GC and BC could be explained in part by the level of familiarity each physician has with patients with GC or BC. Thus, the need to assess baseline cardiac function before starting trastuzumab therapy should be emphasized among specialties that treat patients with GC. Another potential explanation is that chemotherapy combined with trastuzumab varies between BC and GC. For instance, taxanes were typically used in combination therapy for BC, whereas fluoropyrimidine plus oxaliplatin or cisplatin was used for GC.^12^, ^20^ Although some studies reported that docetaxel, one of the taxanes, increased the risk of cardiotoxicity when combined with HER2‐targeted therapy, the precise role of docetaxel to cardiotoxicity remains unclear.^11^, ^21^, ^22^ By contrast, fluoropyrimidine can induce symptomatic or asymptomatic myocardial ischemia, with the incidence rate varying depending on dose and schedule.^11^ However, whether fluoropyrimidine increases the incidence of cardiotoxicity when combined with trastuzumab remains unclear. Although the effect of combination is unclear in both BC and GC, these chemotherapies used in combination with trastuzumab may be related to the baseline UCG performance. Furthermore, we found that the majority of patients with BC were treated by surgeons, whereas the majority of patients with GC were treated by internists. This finding implies that physicians who treat GC and BC are not the same. Trastuzumab may be unfamiliar to the physicians treating GC because it was approved in Japan for HER2‐positive stage IV GC in 2011, 10 years after it was approved for BC. This lack of experience among physicians who treat GC might lead to suboptimal UCG performance before starting trastuzumab therapy. Although a previous study showed that primary care physicians were more likely than oncologists to be unaware of the adverse effects of several chemotherapies,^23^ our findings suggest that oncologists who treated some cancer types were unfamiliar with medications used to treat other cancer types. Further studies are warranted on how to safely use cancer treatments, as targeted therapy will be selected for various types of cancer as the use of precision medicine increases.^24^

In our study, older age was related to UCG performance in all patients and those with trastuzumab therapy. This finding was consistent with previous studies' results.^16^, ^25^ According to an Australian study, older age was related to cardiac function assessment for metastatic BC.^25^ Since guidelines suggested old age as one of the risk factors for cardiotoxicity induced by trastuzumab and anthracyclines,^8^, ^11^ physicians might pay more attention to elderly patients than to young patients. Furthermore, using the Truven Health Analytics MarketScan database, Henry et al. reported that trastuzumab‐induced cardiotoxicity was more prevalent in elderly patients than in young ones.^16^ Recently, the Heart Failure Association of the ESC and the International Cardio‐Oncology Society developed a risk stratification tool to assess baseline risk factors for cardiotoxicity in patients receiving potentially cardiovascular toxic cancer medicines, including anthracyclines and trastuzumab.^26^ This tool has also been validated in patients with early BC who are receiving trastuzumab.^27^ In these studies, several risk factors for cardiotoxicity induced by anthracyclines and trastuzumab were identified, including older age, low baseline LVEF, and previous cardiotoxic cancer treatment. This tool exhibited low sensitivity (14.8%) and high specificity (93.2%),^27^ implying that risk stratification would help identify high‐risk patients and encourage physicians to implement strict guideline‐based cardiac function follow‐up. Because our study findings revealed that baseline cardiac function assessment in anthracycline‐treated patients and patients with GC was inadequate, more rigorous baseline risk stratification should be applied to these patients.

## 5 LIMITATIONS

There are some limitations in this study. First, only UCG performance before starting trastuzumab or anthracycline therapy was evaluated as a test of cardiac function. The use of other tests such as cardiac scintigraphy was not investigated. The focus on UCG may have underestimated the performance rate of cardiac function tests. However, UCG is the most frequently used method to assess LVEF.^28^ Second, only patients who actually received trastuzumab and anthracyclines were included. Patients who had UCG before starting systemic therapy already had cardiac dysfunction and did not receive trastuzumab or anthracyclines were excluded from our study. Nevertheless, we believe that the number of such patients was negligible. Third, data were only acquired from designated cancer care hospitals and nondesignated hospitals that played similar roles to the designated hospitals in their respective community and participated in the quality‐of‐care project. This could lead to selection bias because these hospitals were considered to be highly motivated to improve their clinical practice. Although we found that the UCG performance rate was suboptimal in patients with BC or GC receiving trastuzumab, the UCG performance rate in other hospitals may be lower. Furthermore, we were unable to find cases of UCGs being performed in hospitals other than those where the patients were receiving cancer therapy. Fourth, we conducted statistical tests on many factors and interpreted individually without specific adjustment for multiple comparison, potentially resulting in type 1 error. We must bear in mind the exploratory nature of the analysis. Fifth, there were several concerns about using administrative data to evaluate the quality of care, particularly in the United States.^29^, ^30^ Because only care processes, not results, are coded in the data, administrative claims data do not necessarily capture all aspects of real‐world practice. Nevertheless, we focused on the performance of UCG along with the use of trastuzumab and anthracyclines, our database included sufficient data for this work. When using administrative claims data for quality measurement, data validity is one of the major concerns.^29^ A previous study found that DPC survey data accurately identified procedure records using chart review data as the standard reference.^31^ This result indicated that the quality measure based on DPC data was highly reliable. Sixth, some physicians may choose not to perform baseline UCG on patients with poor life expectancy to minimize patient burden due to comorbidities and aggressiveness, which differ between BC and GC. This might have resulted in an underestimation of UCG performance in this study. Because of the aforementioned possibility of selection bias, it will be difficult to generalize our study findings. Finally, we were unable to assess clinical outcomes such as the development of heart failure or reduced LVEF. Future studies should evaluate the effects of suboptimal UCG performance on clinical outcomes, particularly in patients receiving anthracyclines, and patients with GC receiving trastuzumab. However, to the best of our knowledge, this is the first study to demonstrate suboptimal UCG performance in patients treated with anthracyclines compared with trastuzumab, as well as inadequate UCG performance in patients with GC receiving trastuzumab. Therefore, care must be taken when either well‐known old drugs or newly approved drugs are given, even if they have previously been approved for other cancer types.

In conclusion, data from a large nationwide database revealed that the baseline UCG performance rate varies between trastuzumab and anthracyclines, as well as among cancer types. Interventions for these vulnerable patients are required to improve the quality of care. The cardiotoxicity of anthracyclines, as well as the significance of baseline cardiac examination, should be emphasized. Furthermore, even though medications have already been approved to treat particular cancer types, education on treatment management following approval for additional cancer types will be crucial because of the future development of precision medicine‐based targeted therapy for various types of cancer.

## AUTHOR CONTRIBUTIONS

Taisuke Ishii: Conceptualization, data curation, formal analysis, project administration, writing—original draft, and writing—review and editing. Tomone Watanabe: Data curation, methodology, supervision, and writing—review and editing. Takahiro Higashi: Formal analysis, methodology, software, supervision, and writing—review and editing.

## CONFLICT OF INTEREST

The authors have no conflict of interest to declare.

## ETHICS STATEMENT

This study was conducted as a part of the quality‐of‐care project approved by the Institutional Review Board of the Japanese National Cancer Center. It was conducted following the principles of the Declaration of Helsinki.

## Supporting information


Table S1
Table S2

## Data Availability

Data is used with special permission granted by privacy protection law and data use agreement with the hospitals. They are not publicly available.
