# Supplementary material for: Baseline cardiac function checkup in patients with gastric or breast cancer receiving trastuzumab or anthracyclines
Source: Cancer Med. 2022 Jun 11;12(1):122–30. doi: 10.1002/cam4.4929 (PMC9844617; doi:10.1002/cam4.4929)
Supplement: Supplementary file 1 — Table S1 Table S2 [file CAM4-12-122-s001.docx]

**Table S1.** Characteristics Stratified by Baseline UCG Performance (online only)

| **Characteristics** | **Total**  **(*N* = 6,271)** | **UCG+**  **(*n* = 3,963)** | **UCG−**  **(*n* = 2,308)** | ***P* value** |
| --- | --- | --- | --- | --- |
| Age, mean (SD, min–max), years | 62 (12, 21–92) | 63 (12, 21–91) | 60 (12, 22–92) | <0.001 |
| ≥65, *n* (%) | 2,795 (44.6) | 1,926 (48.6) | 869 (37.7) | <0.001 |
| Gender (female), *n* (%) | 3,910 (62.4) | 2,325 (58.7) | 1,585 (68.7) | <0.001 |
| Cancer type, *n* (%) |  |  |  | <0.001 |
| Breast cancer | 3,246 (51.8) | 1,849 (46.7) | 1,397 (60.5) |  |
| Gastric cancer | 3,025 (47.9) | 2,114 (53.3) | 911 (39.5) |  |
| Treatment type, *n* (%) |  |  |  | <0.001 |
| Trastuzumab | 4,324 (69.0) | 3,105 (78.4) | 1,219 (52.8) |  |
| Anthracycline | 1,947 (31.0) | 858 (21.6) | 1,089 (47.2) |  |
| Treatment duration, median (IQR), days |  |  |  | <0.001 |
| Trastuzumab | 208 (77–398) | 216 (80–407) | 184 (69–368) | 0.001 |
| Anthracyclines | 70 (63–119) | 71 (63–121) | 70 (63–118) | 0.21 |
| Physician specialty, *n* (%) |  |  |  | <0.001 |
| Internal medicine | 2,380 (37.9) | 1,699 (42.9) | 681 (29.5) |  |
| Surgery | 3,891 (62.1) | 2,264 (57.1) | 1,627 (70.5) |  |
| Hospital type, *n* (%) |  |  |  | 0.17 |
| Non-designated hospital | 715 (11.4) | 435 (11.0) | 280 (12.1) |  |
| Designated hospital | 5,556 (88.6) | 3,528 (89.0) | 2,028 (87.9) |  |

Abbreviations: IQR = interquartile range; min, minimum; max, maximum; SD = standard deviation; UCG+, adequate ultrasound echocardiogram performance; UCG−, inadequate ultrasound echocardiogram performance.

**Table S2** Characteristics of Trastuzumab-Receiving Patients Stratified by UCG (online only)

| **Characteristics** | **Total**  **(*N* = 4,324)** | **UCG+**  **(*n* = 3,105)** | **UCG−**  **(*n* = 1,219)** | ***P* value** |
| --- | --- | --- | --- | --- |
| Age, mean (SD, min–max), years | 64 (11, 21–92) | 64 (12, 21–91) | 64 (11, 22–92) | 0.38 |
| ≥65, *n* (%) | 2,337 (54.1) | 1,695 (54.6) | 642 (52.7) | 0.25 |
| Gender (female), *n* (%) | 2,003 (46.3) | 1,482 (47.7) | 521 (42.7) | 0.003 |
| Cancer type, *n* (%) |  |  |  | 0.002 |
| Breast cancer | 1,334 (30.9) | 1,000 (32.2) | 334 (27.4) |  |
| Gastric cancer | 2,989 (69.1) | 2,105 (67.8) | 885 (72.6) |  |
| Trastuzumab duration, median (IQR), days | 208 (77–398) | 216 (80–407) | 184 (69–368) | 0.001 |
| Physician specialty, *n* (%) |  |  |  | 0.08 |
| Internal medicine | 2,221 (51.4) | 1,621 (52.2) | 600 (49.2) |  |
| Surgery | 2,103 (48.6) | 1,484 (47.8) | 619 (50.8) |  |
| Hospital type, *n* (%) |  |  |  | 0.22 |
| Non-designated hospital | 471 (10.9) | 327 (10.5) | 144 (11.8) |  |
| Designated hospital | 3,853 (89.1) | 2,778 (89.5) | 1,075 (88.2) |  |

Abbreviations: IQR = interquartile range; min, minimum; max, maximum; SD = standard deviation; UCG+, adequate ultrasound echocardiogram performance; UCG−, inadequate ultrasound echocardiogram performance.
